# Impact of Li-Ion Battery on System’s Overall Impedance and Received Signal Strength for Power Line Communication (PLC)

**DOI:** 10.3390/s22072634

**Published:** 2022-03-29

**Authors:** Vlad Marsic, Tazdin Amietszajew, Christopher Gardner, Petar Igic, Soroush Faramehr, Joe Fleming

**Affiliations:** Centre for Advanced Low Carbon Propulsion Systems, Institute for Clean Growth and Future Mobility, Coventry University, Coventry CV1 5FB, UK; vlad.marsic@coventry.ac.uk (V.M.); taz.amietszajew@coventry.ac.uk (T.A.); gardne62@uni.coventry.ac.uk (C.G.); petar.igic@coventry.ac.uk (P.I.); soroush.faramehr@coventry.ac.uk (S.F.)

**Keywords:** power line communication, impedance, Li-ion, battery, smart cell, FSV

## Abstract

In anticipation of the hybrid utilisation of the radio frequency (RF) wireless transceiver technology embedded in future smart Li-ion battery cells to deliver hybrid links based on power line communication (PLC) and wireless connections, herein we present an empirical high-frequency investigation of the direct current (DC) bus. The focus is to determine, via statistical tools including correlation coefficient (CC), root mean squared error (RMSE) and feature selective validation (FSV) method, the impedance and signal change impact on a possible communication link when fully charged cells are present or completely missing from the bus. Moreover, to establish if technological differences may be accounted for during the empirical experiments, Li-ion cells from two different manufacturers were selected and connected via three subsequent capacitive couplings of 1 µF, 1 nF and 1 pF. According to a methodical comparison by employing CC, RMSE, and FSV over the measured impedance and signal attenuation, this study has shown that the physical DC network is the dominant impedance at high frequencies and that the signal attenuation on the DC line supports communication in the investigated spectrum. The reported findings are critical for in situ hybrid PLC and wireless communication implementation of BMS for Li-ion systems supported through only one RF transceiver.

## 1. Introduction

### 1.1. Health-Monitoring Technology in Energy Storage

Modern electro-mobility and stationary energy storage require high-energy densities in a small volume and within an accessible budget; therefore, Li-ion rechargeable batteries are in the foreground of current research and development. Nevertheless, these advantages over similar technologies arise with an intrinsic management burden, since the energy storage system’s exposure to unforeseen environmental disturbances, such as vibrations and inertial tensions or the caloric stress of the high-power cycling, may induce thermal runaway or explosion.

A battery-management system (BMS) mitigates these limitations by employing an active monitoring of the battery’s state, such as state of charge (SOC) and state of health (SOH) [1,2,3]. Saving weight and lowering expenses as a result of the cells’ combination into large-scale battery systems demand that the BMS technology focus on power line communication (PLC) [4,5] and wireless solutions [6,7].

The current state-of-the-art research techniques are focused on both mentioned data link alternatives, investigating the limiting factors of physical connections in delivering a robust signal modulation [8] or a high data rate [9], whereas the main concerns for the wireless counterpart are represented by the consistence of the transmitted signal [10] and network integrity [6]. Since recent disseminated evidence is suggesting a possible five-times-faster charging for standard Li-ion cells [11] if cell temperature is controlled while energised [12], or supporting the recent developed fast charging cells [13] by assisting thermal modulation, overcoming the specific barriers in BMS functioning in wired and wireless individual connections may generate substantial benefits.

### 1.2. Enabling the Future over Conventional

**Figure 1:**
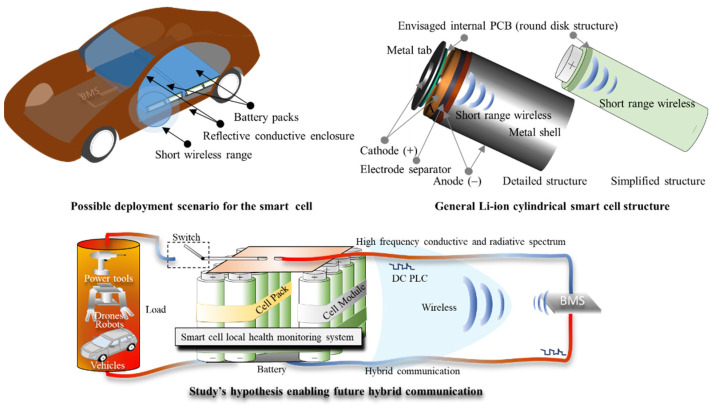
Smart cell via RF transceiver technology introduced inside cylindrical battery shell (**top right**), the resultant densely packed network (**top left**), and the potential for hybrid wired–wireless communications at hierarchical levels utilized in e-mobility platforms (**bottom**).

The current PLC technology is based on wired modems and is separate from wireless technology. Smart homes and industrial automation for long cable distances in AC PLC use classifications that clearly distinguish between the conductive and radiative electromagnetic spectrum from an EMC standpoint [14], while narrowband and broadband are also distinct classes [15,16]. In contrast, these standards [17,18] do not apply to short cable distances used in e-mobility platforms, such as within automotive batteries. Exploiting this unrestricted avenue, a hybrid wired–wireless communication for BMS may naturally result if HF transceivers are used for battery hierarchy access down to cell level. Therefore, the use of free-license RF transceivers such as 868/915 MHz and 2.4 GHz [19] that operate within the required EMC/EMI regulations for short-range devices (SRDs) [20] will not violate any radiation regulations, as branched cables will attenuate rather than amplify the signal. Researchers have found that placing HF transceivers close to power cables will result in direct line coupling, or by using wavelength coupling cable adjustments, hybrid communication trough wires and close vicinity wireless can be achieved [21,22,23]. A large and dense network constructed from cylindrical cells with embedded communication systems inside their metal shells, as illustrated in Figure 1, may find this hybrid capability extremely useful. Through the use of RF transceivers, it can be possible to exploit the conductive paths between the densely packed cells as leaky antennas [24,25] or waveguides [26,27], following obstructions and connecting the hidden nodes found in traditional wireless networks [28]. Additionally, in the event that the power tabs or cables are disrupted or physically damaged, the possibility of diagnosis and wireless communication over short distances will overcome the disconnected node problem found in traditional wired networks [29]. However, proving that HF communication can be a viable option over short distances such in the order of meters through cables connected to a DC line needs to account for cells’ added impedance, voltages and currents that may result from their series and parallel combinations.

### 1.3. Contribution and Paper Organization

Considering that a hybrid solution of using both BMS’s connections by wiring the RF transceivers to the PLC may provide wider advantages than concentrating on each individually, this study will answer through empirical investigation of the DC line response in the HF spectrum, from 1 MHz to 3 GHz, the anticipated question of how viable the RF alternative would be. The experiments will enable us to identify if the system impedance dominant characteristic is imposed by the physical empty line or by the battery cells.

In addition, relying on the same spectrum interval, a sine signal emulating a common data carrier will be utilised to investigate if the signal attenuation on the DC line is sufficient for RF proposed wired solution. The receiver (Rx) sensitivity of −85 dBm similar with the ones operating at 868/915 MHz and 2.4 GHz band [30,31] is used as a reference for line’s link communication available budget. Moreover, the impedance and signal attenuation tests employ two different battery manufacturers for the same Li-ion cell type to determine if the brand may induce a significant difference while conducting identical experiments. 

To unequivocally demonstrate the Li-ion cell’s contribution to the system’s overall impedance and signal attenuation, a variety of combinations must be investigated. The general visual assessment used in various comparison studies for comparing resulted data patterns is undoubtedly insufficient for this investigation; therefore, statistical methods will be employed as an alternative assessment method.

This work delivers an important contribution to the BMS research community by illustrating the premises of HF impedance and signal attenuation measurements in the context of the smart cell integration by wired PLC. Moreover, if the data generated in this study is used as a starting point, the premises of wired–wireless communication inside densely packed networks, such as the battery of an e-mobile platform, may be exploited in future smart cell studies.

**Figure 2:**
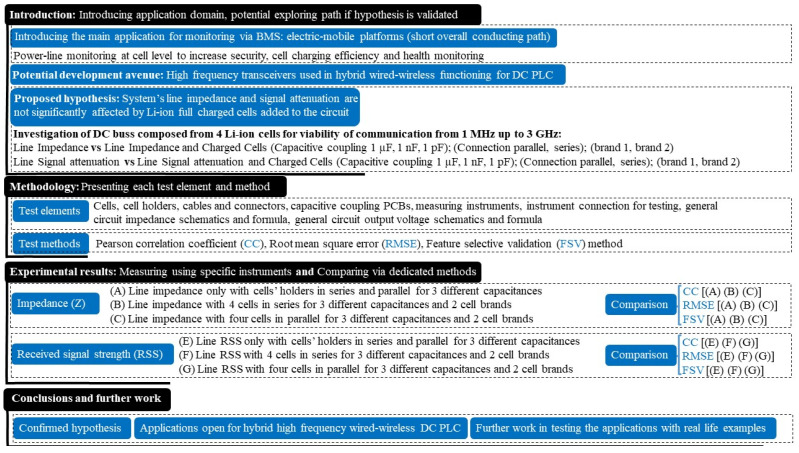
Study’s proposed framework.

The work is structured in four main sections. In the introductory section, the overall topic and suggested technical gap that may allow an effective hierarchical BMS communication solution for e-mobile platforms is presented. The hypothesis that will guide the empirical investigation in the following section is also formulated in the first section. In the second section, we describe each testing element and the statistical tool used to compare the direct measured data streams. While a review of the system’s impedance measurements, position and output voltages is required for the empirical data collection, no numerical or analytical modelling of impedances and voltages will be pursued, since all are based on equivalent simplified circuits. To determine the cell’s influence on the system parameters of interest, the high number of measurement results for each scenario will be compared in pairs in section three. The statistical methods will demonstrate the similarity or dissimilarity of the data, and the fourth section will summarize the findings and state the potential benefits of the work. Figure 2 schematically illustrates the proposed framework for this study.

## 2. Preparation, Experimental Setup and Methodology

### 2.1. Overall Methodology

**Figure 3:**
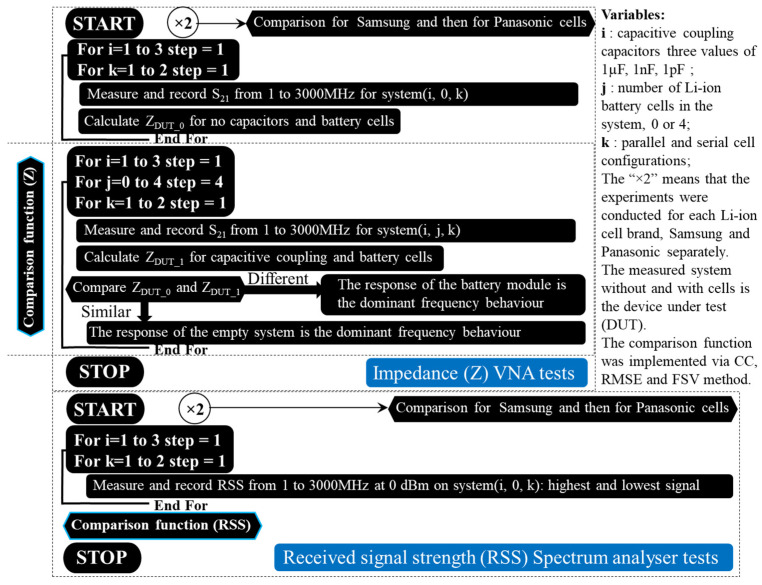
Study’s testing methodology: stages, parameters and operations.

A pseudocode representation of the investigation stages can be found in Figure 3 due to the large number of analysed data used and their different combinations used for comparison. Using the vector network analyser (VNA), the measured scattering parameters (i.e., S parameters) are converted into real impedance values for the interval between 1 MHz and 3 GHz in order to measure the system’s impedance variation with and without Li-ion cells. Since two cell manufacturers are available for testing, each test is conducted subsequently for each brand (i.e., “×2”). In addition, after comparing the series and parallel combinations for all three capacitive coupling values to determine the difference between an empty system and a populated one’s impedance, the same comparison is made between the two cell brands to see if any differences exist. Next, the same algorithm is applied to the received signal strength (RSS), but instead of the VNA’s ports, a signal generator for the transmitter and a spectrum analyser for the receiver are used. In order to illustrate the end limits for the potential budget link calculation, the spectrum interval is also recorded at its maximum and minimum values. Further comparisons are carried out on the RSS averaged values in the same manner as described for the impedance parameter. Next, each testing element and method will be presented briefly, and their selection explained in the study’s context, following the proposed illustrated investigation methodology. 

### 2.2. Selection of the Li-Ion Cell Type Used in the Experiments

Electronic components in situ of a cell provide valuable insight into internal cell state [32], which is advantageous regardless of the cell format. The cylindrical form factor has become widely adapted throughout a variety of industries, due to its attractive power-to-volume ratio, which makes it well suited for various mobile applications, various recent studies concentrating their investigation on this cell type for new sensors for in situ monitoring [33,34]. Furthermore, their form factor provides an undemanding way of organizing custom assemblies due to the standardisation of their dimensions enabling them to be assembled into substantial systems. Additionally, they are preferred in automotive applications [35,36] due to their inherent mechanical protection against external stress factors such as pressure, mechanical impact, acceleration, and braking.

Incorporating electronic circuitry for PLC in situ of an 18650 to create a smart cell is a challenging task due to size constraints and corrosive in-cell environment. If found successful, however, the materials, processes and electronics could be easily transferred to larger cylindrical cell models such as the 21700 type. Studies which have characterised the 18650 model for their experiments have used cells from Samsung [37] and Panasonic [38], or a combination of cells from both manufacturers [39,40]; therefore, in this work, both mentioned cell brands will be used for this study’s experimental work.

### 2.3. DC PLC Experimental Coupling Method

Connecting integrated circuits directly to a high voltage line can damage sensitive semiconductors’ and produce unwanted electromagnetic interference; therefore, a method for safe coupling is required. Such approaches include an inductive, capacitive and hybrid mix; for this study, a capacitive coupling methodology is employed, due to the capacitor’s main property to block DC while maintaining transparency for AC transmission, as demonstrated in recent PLC studies [41,42,43], meaning that it represents a superior bridge option. Therefore, capacitive coupling will be examined from the perspective of line impedance matching for optimum power transfer to the load and matching for minimum noise [44]. However, as neither method of coupling–matching is criticism-free [45,46], it is determined that the most effective solution will be provided by testing on the DC line using three representative capacitor values of 1 µF, 1 nF and 1 pF. To serve this purpose, three PCBs have been created, replacing the breadboard setup with soldered coupling capacitors for improved HF testing. The PCBs used for capacitive DC coupling are symmetrical and measure 10 cm between the input and output connectors.

### 2.4. Battery Cell Holders and Connectors

**Figure 4:**
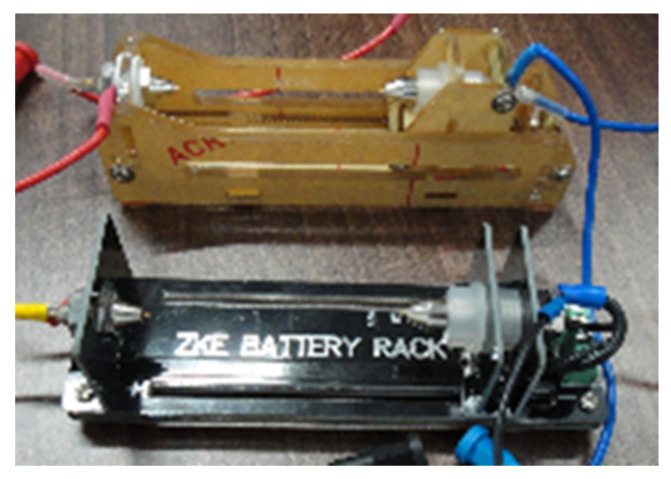
Spring loaded battery receptacles used in experimental research.

For testing different types of cells in certain combinations, welding metal tabs onto the terminals of the plus and minus cells is not an effective solution. In this context, a spring-loaded support would be more appropriate. As a facile method of simplifying the various cells combination and operation, four spring-loaded receptacles [47], interconnected by 1 m cables [48] have been selected for these experiments, Figure 4 illustrating the holder model.

The experiments were performed at room temperature without attaching any loads to the DC bus and avoiding sources of powerful electromagnetic noise. In view of the experiments demonstrating a proof-of-concept phase, changing the above factors is not necessary at this stage. The investigation consists of two separate instrumental setups: one for measuring HF impedances, and another for evaluating the attenuation of signals in the DC system at high frequencies.

### 2.5. The Experimental Setup for Impedance (Z)-Measuring Overview

**Figure 5:**
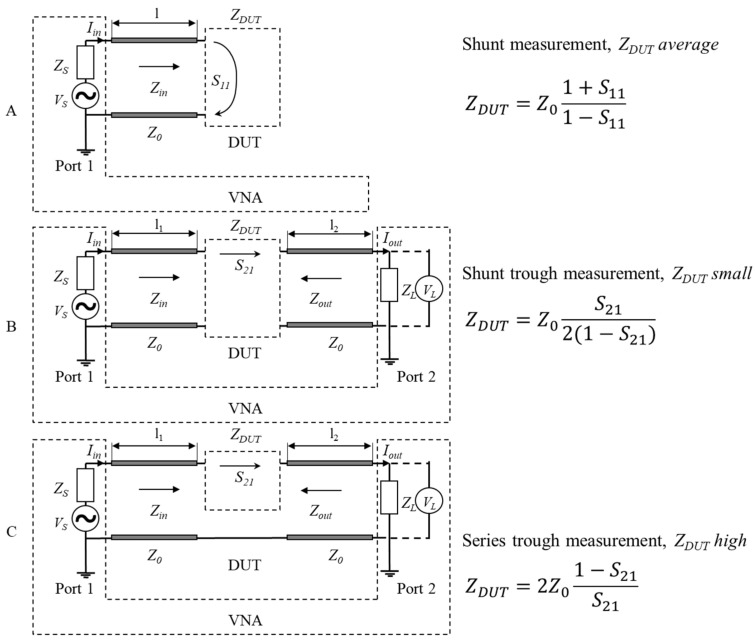
Vector network analyser (VNA) measurements setup for device under test’s (DUT’s) impedance measurements: shunt (**A**); shunt through (**B**); series through (**C**).

For measuring the impedance of the HF DC system, ZNLe3 vector network analyser [49], which covers a spectrum up to 3 GHz, was used with high-quality testers ZV-Z191 model .36 [50]. There are three general configurations for connecting the VNA to the device under test (DUT), depending on the impedance scale to be measured [51,52]. Figure 5 illustrates schematically the three different connections to DUT: the shunt measurement for average *Z_DUT_* A, the shunt trough measurement when *Z_DUT_* is small B and the series trough measurement for high *Z_DUT_* C. 

For this study case, the VNA was connected “shunt-trough” such as illustrated in Figure 5B, as the target impedance is small. It should be noted that the coaxial cable length *l = l*_1_ = *l*_2_ of the VNA probes results in a characteristic impedance *Z* equal to the reference impedance *Z*_0_, which is used in *Z_DUT_* formula. When the cables and setup are combined to result in another characteristic impedance, for instance with a system fixture for a new integrated circuit (IC), the new characteristic impedance can be determined according to [53].

### 2.6. Received Signal Strength (RSS) Attenuation-Measuring Factors Discussion

The experiment work for signal attenuation was carried out using the signal generator SMB100B [54] and a FPC1500 spectrum analyser [55]. This combination simulated the transmitter and receiver on the DC line. The transmitter’s injected signal is connected to the DUT via capacitive coupling, which isolates the DC from the transmitter. However, when the signal reaches the DC zone, it can be considered as an individual voltage source connected to a battery network.

**Figure 6:**
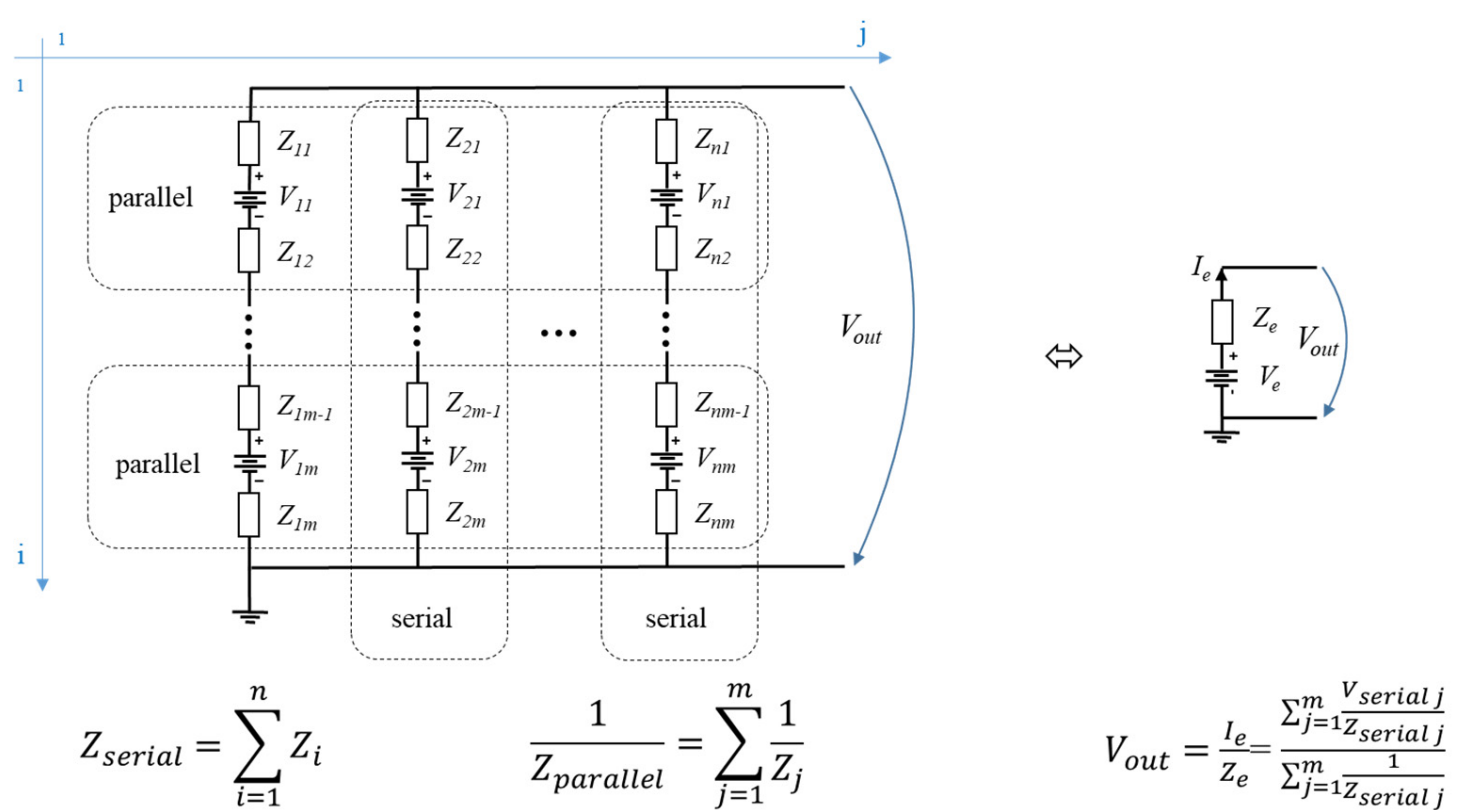
Output voltage of a battery network connected in series up to n and parallel up to *m*, transforming first by summing on each column the serial equivalent voltage, currents and impedances, then summing the equivalent voltages, currents and impedances for parallel configuration on the remaining row.

A battery network is one example of a voltage-summating mesh, in which the DC power sources interact with the impedances in series or parallel arrangements. Figure 6 shows a generalised view of a battery network, along with the equations for output voltage and impedance implicitly calculated for the equivalent resulted circuit’s output voltage.

### 2.7. Data Analysis Methods

Three unique comparison methods were selected for analysing the empirical data records: correlation coefficient (CC), root mean square error (RMSE) and feature-selective validation (FSV). The Pearson correlation function [56,57] is one of the standard correlation functions used in statistical research, and it measures correlation between two data sets on a closed interval of [−1, 1], where ±1 signifies perfect correlation and zero means no match. As the second tool, RMSE, shows the absolute difference between data values, the best data fit between two signal streams will yield a RMSE of zero [58]. Generally, the method is used to analyse differences between real measurements and simulation data sets, the non-null difference indicating that model improvements are still necessary. A third additional method was appointed to cover methodical testing of relational data for a less conceptual and more comprehensive representation. For this study case, since the test data concerns electromagnetic measurements, the feature selective validation method [59,60], found in IEEE standard 1597.1 [61], was selected.

The FSV method allows evaluation at two stages, utilizing empirical data filtering and grouping. In the first step of the method, the frequency domain comparison data is divided into DC, low-frequency (Lo) and high-frequency (Hi) intervals. The breaking point between Lo and Hi is N, which is set at 40% of the sum value of the independent variable. As a next step, the method introduces the first level of agreement testing, for the data now converted back in the time domain, by utilising an amplitude-difference measure, (ADM). The second level of agreement testing, the feature-different measure (FDM), is then calculated based on the first- and second-order derivatives to determine the data features for the Lo and Hi data sets. In the end, this method computes a third value using the previous two, which is the global difference measure (GDM), and which provides a measure of “goodness-of-fit”. The ADM, FDM and GDM provide quantitative visual interpretations for data similarity and map a quality interpretation on six intervals, as shown in Table 1. FSV has its advantages and limitations, as do all other statistics methods [62]. In contrast to classical statistical and algebraic toolsets, this method is relatively new and there has been active research conducted to eliminate its limitations and extend its applicability to two-dimensional (2D) and three-dimensional (3D) data sets [63].

### 2.8. Experimental Bounds

A small impedance difference is expected to be introduced into the grid when Li-ion batteries are present, indicating the line network has the dominant impedance. Furthermore, the difference between the cells from the two different manufacturers is expected to be small, demonstrating that the experiment can be translated to other cells produced to similar standards. As stated previously, no external factors causing variations to the DC line were considered except the test signal, therefore the presumption is that the experimental signal attenuation should relate to the physical property of the DUT, and it is expected that the receiving signal strength (RSS) level will be higher than −85 dBm, which is typically the threshold for the selected reference receiver sensitivity.

## 3. Experimental Results and Discussion

### 3.1. Impedance Measurement

**Figure 7:**
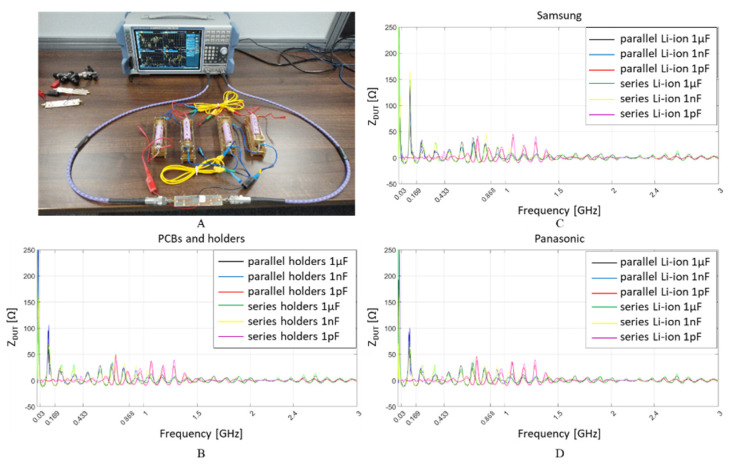
VNA impedance measurements setup (**A**); for 1 µF, 1 nF and 1 pF capacitive coupling to: the empty system (i.e., no Li-ion cells present inside holders) (**B**); four Li-ion Samsung 18650 cells connected in parallel and series (**C**); four Li-ion Panasonic 18650 cells connected in parallel and series (**D**).

Illustrated in Figure 7A is the measurement setup and the impedance plots for the empty system Figure 7B, added four Samsung cells in Figure 7C and the same combinations for four Panasonic cells in Figure 7D. According to the series-parallel configurations for the three coupling capacitances, the impedance results are divided into six categories for each plot.

System impedance changed slightly with and without Lithium-ion cells, between 1 MHz and 3 GHz. In the low frequency range (i.e., below 10 MHz), there is a high impedance spike, however, it is scaled down to about 0.25 kΩ to provide visual details of variation for the remainder of the interest spectrum from 30 MHz to 3 GHz. Although the voltages in the system vary from 0 V, when no cells are present, to 3.7 V when cells are connected in parallel, and to 14.8 V when the cells are connected in series, the system’s impedance does not appear to change significantly.

### 3.2. Impedance Data Analysis

The FSV method was applied to the empirical data, produced using the same capacitive coupling setup, to observe the subtle impedance differences between systems with and without the 18650 Li-ion batteries: connections to an empty system versus the same PCBs attached to four Samsung and separately to four Panasonic cells, first in parallel, then in series.

The FSV bar graphs were modified on the Y axis to show the percentage of samples that are recorded from the total data values during one measurement instead of the classical quantitative intervals from zero to three. The ratio provides a better picture of how many exhibit a good agreement (e.g., excellent, very good, good), a neutral one (fair), or no relationship at all (e.g., poor, very poor).

**Figure 8:**
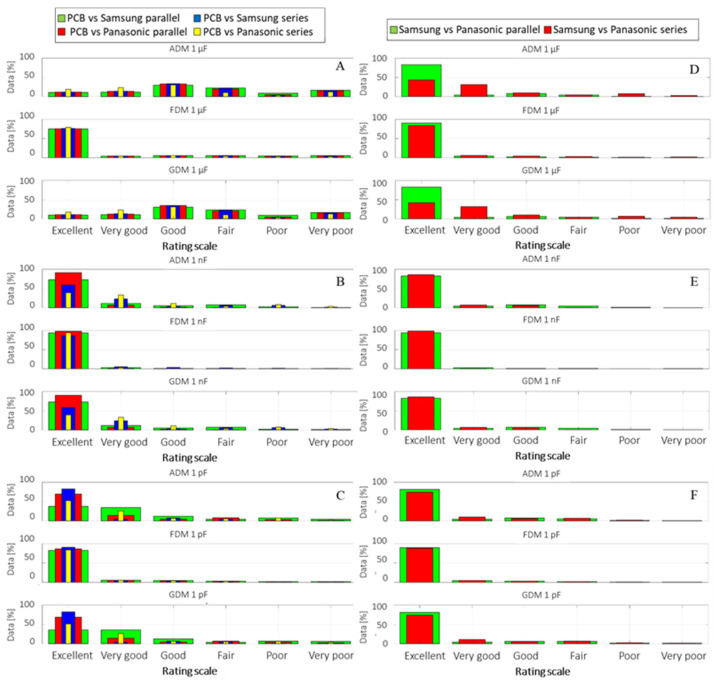
VNA impedance measurements, for 1 µF, 1 nF and 1 pF capacitive coupling to: the empty system (i.e., no Li-ion cells present inside holders) (**A**); four Li-ion Samsung 18650 cells connected in parallel and series (**B**); four Li-ion Panasonic 18650 cells connected in parallel and series (**C**). The impedance comparison between the two different cell brands connected in series and parallel for the three capacitive couplings is illustrated in the (**D**–**F**) plots.

In Figure 8, FSV comparison graphs are shown between the empty system with holders connected in parallel and series, and the full system with four cells from each manufacturer at the time, with a capacitive coupling of 1 µF in Figure 8A, 1 nF in Figure 8B, and 1 pF in Figure 8C, whereas a similar comparison is shown on Figure 8D–F only between the Samsung and Panasonic cells.

It is noteworthy that while all setups show a good data similarity, the one connected through the 1 µF capacitors presents the most variations on the ADM amplitude graph, while the measured signal trends described by the first and second derivatives on FDM show an excellent match between data tendencies. The overall GDM pattern looks as though it inherits the same tendency described by ADM rather that representing an addition of both ADM and FDM; however, when ADM shows variations in the data sample ends (low and high frequency) which are attenuated in FDM, for two data series where the main difference is characterized by the sample domains’ ends, such as in this case, the GDM is reflected accordingly. Similarly, for the 1 pF analysis on C, despite displaying a dominant tendency of 40% “excellent” data correlation, the overall measurement comparison indicates a good data match when combined with the “very good” and “good” ratings.

The FSV agreement between measured impedances for Samsung and Panasonic D to E was found to be very good to excellent, whereas the cell series connection data showed more noise than for the parallel case. When analysing the same data based on the overall indicators, the correlation coefficient and RMSE as shown in Table 2, it can be observed that correlation is higher for the 1 nF and 1 pF couplings and the RMSE is minimum with these intervals, confirming the FSV report.

A lower cut-off frequency caused by 1 µF capacitive coupling may explain the lower data agreement with the two other coupling cases (i.e., three and six orders lower). At this frequency, other external distinctive factors may manifest themselves such as the natural resonant frequencies of the wires interconnecting the battery holders, battery cells, etc. A similar comparison investigated with the correlation coefficient and RMSE, in Table 3, indicates the same as FSV: high correlation between the data sets, with a minimum difference evidenced by the RMSE small values.

Based on the comparison it can be concluded that the DC physical system’s impedance at high frequencies, between 1 MHz and 3 GHz, provides the dominant response, while the 18650 Li-ion cells have an overall small contribution. When comparing cells from different manufacturers in the same DC system, no significant differences were detected.

### 3.3. Received Signal Strength Measurement

**Figure 9:**
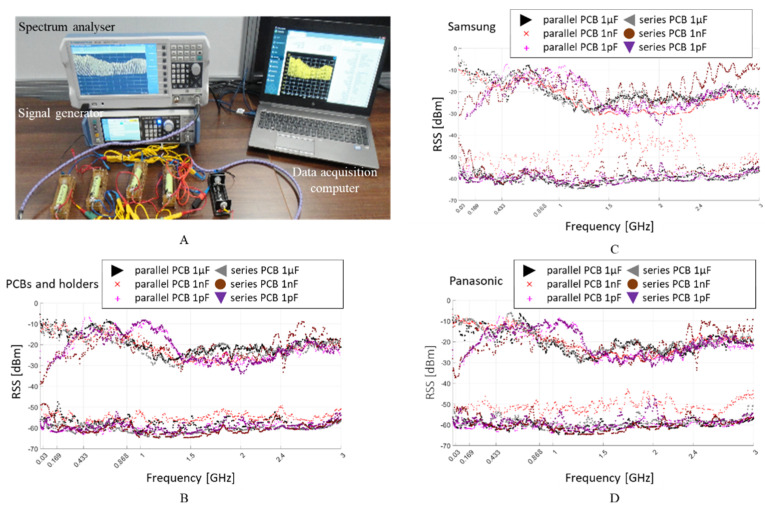
Spectrum analyser received signal strength (RSS), attenuation measurements setup (**A**), for 1 µF, 1 nF and 1 pF capacitive coupling to: the empty system (i.e., no Li-ion cells present inside holders) (**B**); four Li-ion Samsung 18650 cells connected in parallel and series (**C**); four Li-ion Panasonic 18650 cells connected in parallel and series (**D**).

Illustrated in Figure 9A, the maximum and minimum attenuation of a sine wave signal injected into the system by the signal generator and received by the spectrum analyser is measured on the empty battery holders and connectors Figure 9B, Samsung cells Figure 9C and Panasonic cells Figure 9D.

It can be observed that the maximum attenuation is approximately −65 dBm, which is 15 dB lower than the Rx sensitivity of a typical low power RF transceiver designed for 868 MHz or 2.4 GHz. This high RSS attenuation may be attributed to intermediate frequencies that have no activity observed when the spectrum analyser sweeps from 1 MHz to 3 GHz. Up to 1.5 GHz, RSS shows a good reception with just a 10 dB attenuation, while the attenuation increases to 20 dB for higher frequencies forward.

The interval displayed between the RSS max and min plotted limits is between −30 dBm and −50 dBm and provides a very convenient communication range for the 868 MHz and 2.4 GHz based RF transceivers. In the wireless data transfer, when the instant RSS is found in the abovementioned interval, it indicates a strong connection within a short distance between the transmitter and receiver.

### 3.4. Received Signal Strength Analysis

**Figure 10:**
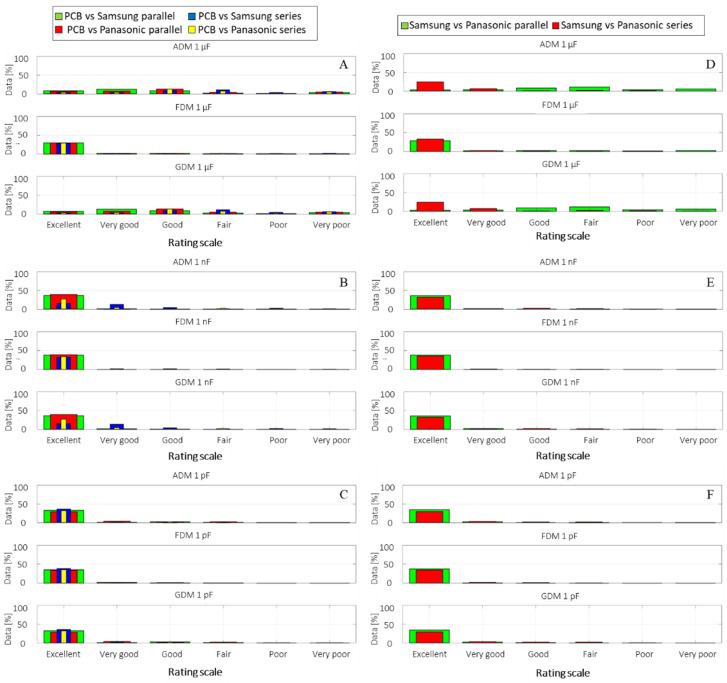
FSV RSS comparison between PCBs of 1 µF, (**A**), 1 nF (**B**), and 1 pF (**C**), capacitive coupling connected to the empty system (i.e., no Li-ion cells present inside holders) and the same system containing Li-ion battery cells from Samsung and Panasonic, respectively, in parallel and series with four cells at the time (**D**–**F**).

Although the minimum and maximum RSS on the HF interval of 1 MHz to 3 GHz have been shown to support the hypothesis of wire communication by means of wireless transceivers at 868 and 2400 MHz, the differences induced by the DC voltages over the injected signal can be analysed via CC, RMSE and FSV analogous with the previous case of system’s impedance. The analysis is carried out in the same manner as the one for the impedance of the different systems by comparing the RSS result from each empty system with that from the system where Li-ion cells from both manufacturers are inserted in parallel and series, Figure 10A–C, whereas comparisons between the two cell manufacturers are shown in Figure 10D–F.

The FSV bar graph comparison reveals an almost similar result as for the VNA measurement setup, reinforcing the consistency of the experimental setup and measurements. With respect to the previous observation regarding ADM low indications for the 1 µF coupling when compared with 1 nF and 1 pF (i.e., similarly for impedance measurements), the ADM scale results are showing an approximate agreement level of 15–20% between the first four ratings: Excellent, Very good, Good and Fair. This may indicate that although both data series are following the same graphical trend, their scale values may be sifted.

Assuming all four ADM ratings are summed, an overall fit of approximately 60–80% can be considered good, but we must keep in mind that data show a higher distributed variance than in the other instances.

According to the CC and RMSE results, the same picture of good-to-very-good data agreement is shown: good for 1 µF coupling, and very good for 1 nF and 1 pF in the first case when the reference system is empty Table 4. In Table 5, the comparison between the two cell manufacturers, at a slightly smaller scale than in the impedance analysis, highlights the interchangeability of the cells.

The evidence obtained from FSV, correlation coefficient and RMSE analysis of the injected signal on the DC PLC composed of four 18650 Li-ion cells suggests that the DC voltages have a small influence on the communication at high frequencies between 1 MHz and 3 GHz. Additionally, differences in voltage level were similar between the two studied battery cell manufacturers, Samsung and Panasonic.

### 3.5. Overall Notes

An element of interest has been noticed as a result of the subsequent changes in coupling capacitances during the measurements and comparisons. When compared to the 1 nF and 1 pF, the 1 µF capacitive coupling is exhibiting a different behaviour. The evidence was noticed when statistical tools were used to compare the data streams, however, on closer inspection when examining the max RSS measurements, it is seen that the dissociating trend acts as the overall system’s cut-off frequency, with a threshold around 1.5 GHz. In this case, it cannot be explained by setup variations since one should experience nonuniform variations, that are not interval-specific-based. According to the microstrip line and strip-line filter theory, the phenomenon may arise from a design flaw in the connecting PCB, as the microwave frequencies investigated here may couple to the copper lines. Accordingly, based on this study’s empirical findings, a more concentrated effort may be useful in future managing the HF signal injection at the board design level.

## 4. Conclusions and Further Work

The present study provided a comprehensive measurement of system impedance and signal strength in three combinations of capacitive couplings and DC voltages added to the lines using four 18650 Li-ion fully charged cells from two different manufacturers. Moreover, by employing the statistical tools delivered through CC, RMSE and FSV method, this empirical work has been methodically compared in pairs while subsequently changing the capacitive coupling capacitances of 1 µF, 1 nF and 1 pF, and the two cell brands, the system impedance and the received signal strength for:

- System containing no cells with the system containing four full charged cells connected in parallel;
- System containing no cells with the system containing four full charged cells connected in series;
- System containing cells from first brand with the system containing cells from second brand, all in parallel configuration;
- System containing cells from first brand with the system containing cells from second brand, all in series configuration.

The comparison results strongly indicate that the Li-ion charged cells from both brands are not contributing significantly to the overall system’s impedance or RSS attenuation, and that the physical system parameters represent the dominant measure when there are no loads or external noises. The initial hypothesis is confirmed that the cells’ contribution to the overall system can be overlooked, since it is minor in comparison with the overall system’s physical connections. Consequently, a possible hybrid wired–wireless network that utilizes HF RF transceivers embedded inside short conductive paths DC networks such as those used in rechargeable batteries for e-mobility platforms is highly plausible if operating over 1 MHz up to 3 GHz interval.

On the basis of min and max RSS measurements, the results suggest that DC PLC communication is possible in the interval of 1 MHz to 3 GHz, suffering a manageable attenuation at high frequencies such in the region for 868 MHz and 2.4 GHz, where the general receiver sensitivity is higher than −85 dBm, depending on the data rate and frame-security-added overheads. Since the maximum signal attenuation encountered was down to 65 dB, a 15 dB margin to account for possible induced noises and signal variation due intermittent loads would be advised in real-life deployment.

Finally, no significant difference at the system impedance and RSS voltage level was found between the tested cell models. Based on the FSV, CC and RMSE analysis, they can be considered close to interchangeable. Further work exploiting the positive results of this study may include comparison cases of wired RF transceivers, wireless transmission experimentation at cell level or a hybrid method that can mitigate network discontinuities or disrupted communication protocols providing high redundancy and reliability.

By illustrating the premises of HF impedance and signal attenuation measurements in the context of smart cell integration, this paper makes an important contribution to the BMS community. In conclusion, the HF impedance and RSS PLC measurement methodology presented in this study may support innovation in both performance and operational cell communication, as well as RF modelling for energy storage systems.

## Figures and Tables

**Table 1:** FSV interpretation scale.

FSV Value (Quantitative)	FSV Interpretation (Qualitative)
value ≤ 0.1	Excellent
value є (0.1, 0.2]	Very good
value є (0.2, 0.4]	Good
value є (0.4, 0.8]	Fair
value є (0.8, 1.6]	Poor
value > 1.6	Very poor

**Table 2:** Correlation and RMSE for impedance measurements comparison between Samsung and Panasonic Li-ion cells, in parallel and series configurations, connected through three different capacitive couplings.

18650 Li-Ion Cells Impedance Z Comparison: Samsung vs. Panasonic						
Configuration	Parallel	Series	Parallel	Series	Parallel	Series
**Capacitors**	**1 µF**		**1 nF**		**1 pF**	
**Correlation**	0.98	1	1	1	1	1
**RMSE**	0.08	0.02	0.02	0.01	0.01	0.01

**Table 3:** Correlation and RMSE for impedance measurements between PCBs (i.e., empty system) and Samsung respectively Panasonic Li-ion cells, in parallel and series configurations.

18650 Li-Ion Cells Impedance Z Comparison: PCBs vs. Samsung A, PCBs vs. Panasonic B												
Configuration	Parallel A	Parallel B	Series A	Series B	Parallel A	Parallel B	Series A	Series B	Parallel A	Parallel B	Series A	Series B
**Capacitors**	**1 µF**				**1 nF**				**1 pF**			
**Correlation**	0.35	0.35	0.46	0.46	0.7	0.7	0.85	0.86	0.99	0.99	0.98	0.97
**RMSE**	1.07	1.08	1.08	1.08	0.19	0.18	0.14	0.14	0.02	0.02	0.03	0.03

**Table 4:** Correlation and RMSE for RSS measurements comparison between Samsung and Panasonic Li-ion cells, in parallel and series configurations, connected through three different capacitive couplings.

18650 Li-Ion Cells Recived Signal Strength RSS Comparison: Samsung vs. Panasonic						
Configuration	Parallel	Series	Parallel	Series	Parallel	Series
**Capacitors**	**1 µF**		**1 nF**		**1 pF**	
**Correlation**	0.80	0.81	0.84	0.78	0.81	0.79
**RMSE**	0.82	0.82	0.58	0.87	0.80	0.82

**Table 5:** Correlation and RMSE for RSS measurements between PCBs (i.e., empty system) and Samsung Panasonic Li-ion cells, respectively, in parallel and series configurations.

18650 Li-Ion Cells Recived Signal Strength RSS Comparison: PCBs vs. Samsung A, PCBs vs. Panasonic B												
Configuration	Parallel A	Parallel B	Series A	Series B	Parallel A	Parallel B	Series A	Series B	Parallel A	Parallel B	Series A	Series B
**Capacitors**	**1 µF**				**1 nF**				**1 pF**			
**Correlation**	0.81	0.82	0.83	0.83	0.85	0.81	0.83	0.86	0.78	0.84	0.85	0.78
**RMSE**	0.78	0.78	0.82	0.78	0.65	0.71	0.80	0.75	0.86	0.77	0.71	0.81

## Data Availability

The dataset generated and analysed during this study is available from the corresponding author on a reasonable request, but restrictions apply to the commercially confident details.
